# Alpine ethnobotany in Italy: traditional knowledge of gastronomic and medicinal plants among the Occitans of the upper Varaita valley, Piedmont

**DOI:** 10.1186/1746-4269-5-32

**Published:** 2009-11-06

**Authors:** Andrea Pieroni, Maria Elena Giusti

**Affiliations:** 1 University of Gastronomic Sciences, Via Amedeo di Savoia 8, I-12060 Pollenzo/Bra, Italy; 2 Dipartimento di Storia delle Arti e dello Spettacolo, Università degli Studi di Firenze, Via Gino Capponi, I-50121 Firenze, Italy

## Abstract

A gastronomic and medical ethnobotanical study was conducted among the Occitan communities living in Blins/Bellino and Chianale, in the upper Val Varaita, in the Piedmontese Alps, North-Western Italy, and the traditional uses of 88 botanical taxa were recorded. Comparisons with and analysis of other ethnobotanical studies previously carried out in other Piemontese and surrounding areas, show that approximately one fourth of the botanical taxa quoted in this survey are also known in other surrounding Occitan valleys. It is also evident that traditional knowledge in the Varaita valley has been heavily eroded. This study also examined the local legal framework for the gathering of botanical taxa, and the potential utilization of the most quoted medicinal and food wild herbs in the local market, and suggests that the continuing widespread local collection from the wild of the aerial parts of Alpine wormwood for preparing liqueurs (*Artemisia genipi, A. glacialis*, and *A. umbelliformis*) should be seriously reconsidered in terms of sustainability, given the limited availability of these species, even though their collection is culturally salient in the entire study area.

## Background

In recent years, only a few ethnobotanical researches in the Alps have focused on the interaction between plant resources and human societies within ethnic minority groups [1-6]. This is regrettable because comparative and/or quantitative analyses aimed at increasing the understanding of how Traditional Knowledge (TK) of botanicals changes across cultures and over time and space are essential if we are to form strategies aimed at sustaining bio-cultural diversity and unique experiences of interactions between nature and cultures, and their transmission mechanisms along generations [7-13].

Furthermore, the Alps represent one of the most interesting but least studied regions in Europe, particularly with regard to the exploration of tangible and intangible cultural heritage related to TK of plants, even though the potential outputs of such researches could be important in sustainability projects focused on organic farming, home gardens, local foods, eco-tourism, eco-gastronomy, and eco-museology; and even though climate change may have a tremendous impact on Alpine biodiversity and related ethnobotanical resources [14].

Although a number of ethnobotanical inventories were compiled during the past 50 years in a few other Occitan [15-20], Franco-Provencal and Walser Alpine [21-23] valleys in Piedmont and the surrounding areas [24-26], no field ethnobotanical studies had been conducted in the Varaita valley. Instead only a few linguistic [27] and ethnographic researches [28-32] took place.

The aims of this study were therefore:

• to record the food and medical ethnobotanical knowledge of the Occitans living in the upper Varaita valley (Chianale and Bellino/Blins);

• to compare the collected data with those available in the ethnobotanical literature of other Alpine valleys in Piedmont and surrounding areas (Aosta Valley, and the French and Swiss sides of the Alps), and to point out the eventual occurrence of a specific "Occitan" ethnobotany;

• to suggest local plant resources of particular interest for sustainable small-scale agricultural/gathering activities and eco-tourism activities.

## Methods

### Ethnographic, historical, and environmental context

The presence of a single "Occitan" culture/language is one of the most disputed issues in European ethnolinguistics [33], given the considerable heterogeneity of the vast area covered by all linguistic varieties linked under the "Occitan" umbrella. In Italy, "Occitan" languages are spoken in a dozen of valleys in the Western Alps in the Piedmont region [34], where the interest of the media has been concentrated in recent years on tracing this disappearing culture [35], and within a small enclave in Southern Italy (i.e. Guardia Piemontese in the Calabria region).

**Figure 1:**
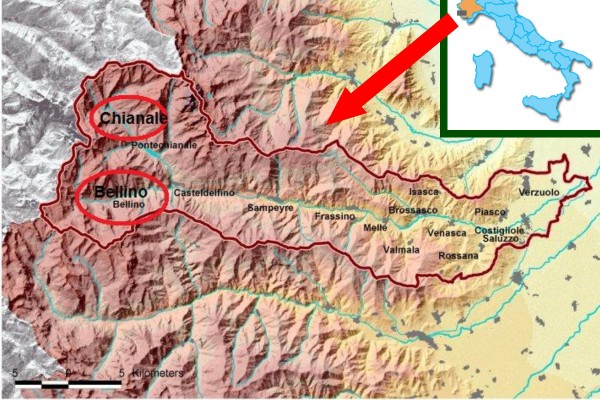
**Location of the study area**.

The focus of this study, the Varaita valley, is located in the Western Alps in the Piedmont region, Cuneo province, in North-Western Italy (figure 1). The valley is crossed by an homonymous alpine torrent, which is 75 km long and springs from the slopes of the mountain, Monviso, in the Cottian Alps near the French border, and enters the Po River near Casalgrasso. From the beginning of the 12th century until 1601, most of the Varaita valley was part of the small but influential marquisate of Saluzzo. The upper Varaita valley, called Chastelada, had a different fate however. For four centuries, from 1343 until 1713, it belonged to an independent federation known as Escartons, which was based in Briançon in France. This federation was a good example of enlightened and progressive self-government [28]. After 1713, the upper valley had a quite complex history due to its location on the border of Italy and France. It was the locus of continuous military incursions in the 18^th ^Century from the French side, and in the 19^th ^and most of the 20^th ^century it saw much intensive smuggling activity.

**Figure 2:**
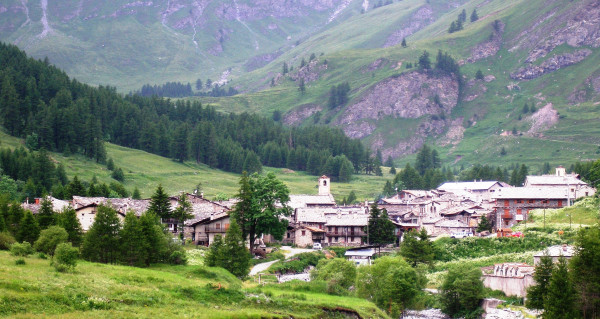
**Chianale**.

At present, only mainly elderly persons still live year round in the upper part of the Valley. Bellino (1480 m a.s.l.) includes the hamlets of Çelle, Chiazale, Chiesa Fontanile, Pleyne, Ribiera, and Sant'Anna, and has the status of a municipality. Chianale (1797 m a.s.l., figure 2) is a hamlet in the municipality of Pontechianale. The hamlets of Bellino and Chianale are among the smallest Alpine villages in Italy (numbering approx. 15-30 permanent, mainly elderly inhabitants in each village).

Regarding vegetation, the upper Varaita valley presents mainly the association *Rhododendro-Vaccinietum cembretosum *(characterized by *Rhododendron ferrugineum*, *Vaccinium myrtillus *and, to a lesser extent, *V. vitis-idaea*, *V. uliginosum*, *Lonicera coerulea, L. alpigena, L. nigra, Juniperus nana, Rosa pendulina, Daphne mezereum, Clematis alpina, Sedum anacampseros, Luzula sieberi, Festuca flavescens and Homogine alpine*), and the subassociation *Junipero-Arctostaphyletum cembretosum *(characterized by *Juniperus nana, Arctostaphylos uva-ursi, Cotoneaster integerrimus, Avenella flexuosa, Vaccinium myrtillus, Minuartia laricifolia, Festuca flavescens, Galium obliquum and Brachypodium caespitosum*). The total rainfall is 854 mm/yr (Casteldelfino, 1296 m a.s.l.); the climate is sub-littoral alpine, with some continental characteristics [36].

The local economy of the upper valley is based on traditional agro-pastoral activities (cow breeding), but this is carried out in only a minor way. There is some tourism, especially in the summer (alpine walking and trekking). Almost all of the few middle-aged and younger members of the community are employed in the lower part of the valley. Traditional cultivation of local staples (rye, barley, buckwheat) had disappeared by the 1970's and home gardens these days are managed as a secondary activity only.

### Field study

**Figure 3:**
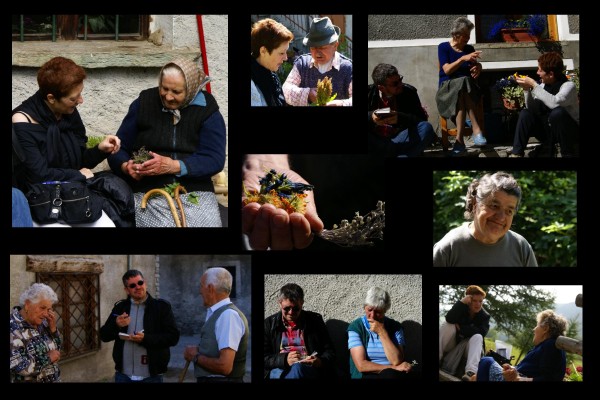
**Documentation of a few interviews conducted during the field study (photo: Nicola Robecchi)**.

Ethnobotanical investigations of the upper Varaita Valley (figure 1), in the communities of Bellino (Celle, Prafauchier, Chiazale, Chiesa, Pleyne, Ribiera, and Sant'Anna, and Bals) and Chianale (figure 2) were carried out over four weeks in July 2008. Local informants (n = 67) were asked about local food and medicinal plants via open and semi-structured interviews (figure 3). Detailed information was sought about the plants' vernacular names, and particularly their past and present uses.

Prior informed consent (PIC) was obtained verbally before commencing each interview, and ethical guidelines adopted by International Society of Ethnobiology [37] were followed. The quoted botanical species were identified using Pignatti's *Flora d'Italia *[38] (with the help of a recent standard work on Alpine flora [39]), and systematically framed as suggested by the most recent checklist of Italian vascular flora [40]. Voucher specimens of the wild taxa were collected, and they are now deposited at the Herbarium of the University of Gastronomic Sciences.

### Data analysis

The data collected during the field study were reported using Microsoft^® ^Excel, and a descriptive comparison was conducted with ethnobotanical data available in the scientific literature (e.g. field studies previously conducted in Piedmont and other surrounding alpine areas [15-26]). However, given the tremendous heterogeneity of the methodological approaches adopted in all these previous studies (in which the number of informants was rarely reported, and in a few cases even the names of the villages visited were omitted), it was impossible to carry out a proper statistical analysis aimed at capturing similarities between our data and the data occurring in the ethnobotanical literature (i.e.: correspondence analysis, Jaccard index).

## Results

All the quoted botanical taxa (n = 88), including their related recorded local names and traditional gastronomic and/or medicinal uses, are reported below. We have included in brackets the voucher specimen codes and the quotation index (+++: taxa quoted by at least 40% of the informants; ++: taxa quoted by more than 10% and less than 40% of the informants; +: taxa quoted by fewer than 10% of the informants).

**Figure 4:**
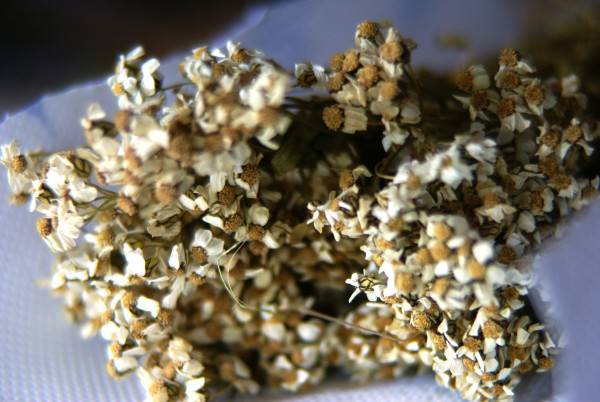
**Dried flowering tops of *Achillea herba-rotta***.

*Achillea herba-rotta *All. (Asteraceae). Wild. Canamìo, Rua. Dried flowering aerial parts (figure 4): tea or macerated in alcohol as a digestive, for treating toothache and headaches, as a mild tranquillizer, or an emmenagogue. Topical application with butter and heated paper, which is then covered with a piece of woollen cloth for treating bad coughs. (CHI-CAN) +++

*Achillea millefolium *L. (Asteraceae). Wild. Ervo taiouro. Dried flowering aerial parts: tea as an anti-haemorrhoidal (humans) or an anti-diarrheic (cattle). Fresh aerial parts: topically applied as a cicatrizing agent. (CHI-ETA) ++

*Alchemilla vulgaris *(Rosaceae). Wild. Gunnellettes (plural). Dried leaves: use not recalled. Traded in the past. +

*Allium cepa *L.(Alliaceae). Cultivated. Seoulo. Fresh bulbs: consumed in salads and as a seasoning in the local cuisine. +++

*Allium porrum *L. (Alliaceae). Cultivated. Por. Fresh whole plant: traditionally consumed mixed in a kind of maize polenta. +

*Allium sativum *L. (Alliaceae). Cultivated. Ai. Fresh bulbs: as a seasoning in the local cuisine (rare). In the past made into necklaces and worn by children as a treatment for worms. ++

*Allium schoenoprasum *L. (Alliaceae). Wild. Poureto. Fresh leaves: added in pancakes (*bignes*) as a seasoning. To be avoided by cows and sheep, as the milk takes on a horrible taste. (CHI-POU) +++

*Antennaria dioica *(L.) Gaertn. (Asteraceae). Wild. Piot de chat. Dried flowering aerial parts: tea as a digestive. Traded in the past. +

*Arnica montana *L. (Asteraceae). Wild. Strugnarelo. Dried flowers: macerated in alcohol or in oil and applied externally for treating bruises. Traded now and in the past. (CHI-STR) +++

*Artemisia absinthium *L. (Asteraceae). Wild. Erbio bioncho, Ousenzo. Dried aerial parts: put in cattle stables and in wardrobes as an anti-parasitic or to drive away moths. Tea used as an anti-hypertensive, digestive, and an antihelminthic (for children). (CHI-BIO) +++

**Figure 5:**
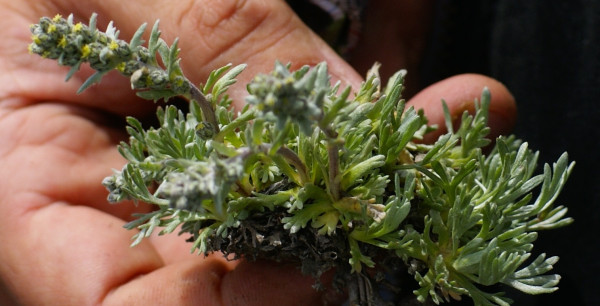
***Artemisia genipi***.

*Artemisa genipi *Weber (Asteraceae) (Figure 5). Wild. Genepì maschi, Genepì nero. Dried flowering aerial parts: tea as a panacea (especially against coughs and as a digestive). Macerated in alcohol as a digestive. Traded in the past and now (illegally). (CHI-GEM) +++

**Figure 6:**
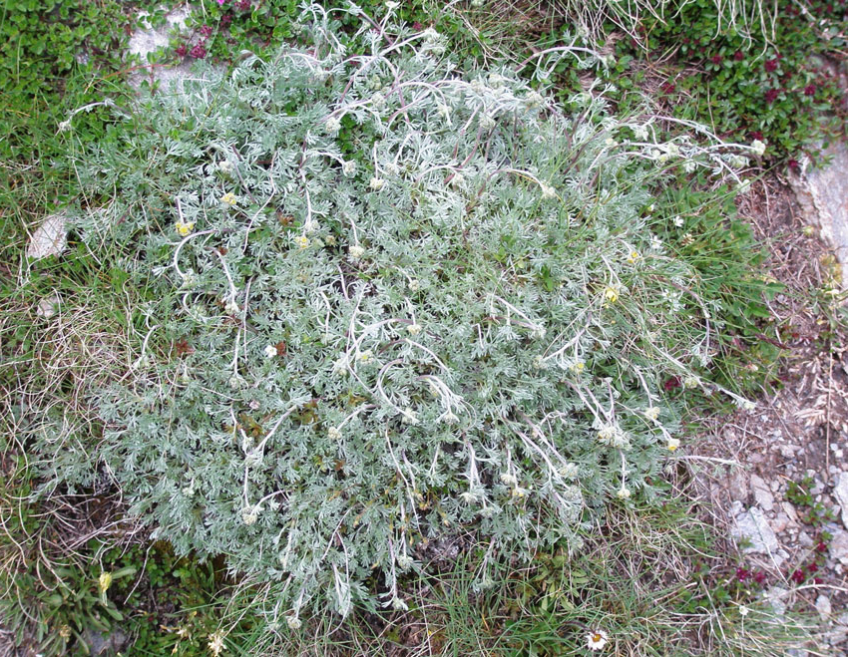
***Artemisia glacialis***.

*Artemisia glacialis *L. (Asteraceae) (Figure 6). Wild. Genepì fumelo, Bava dei ghiacciai. Dried flowering aerial parts: tea as a panacea (rare). Macerated in alcohol as a digestive. Traded now and in the past. (CHI-GEF1) +++

**Figure 7:**
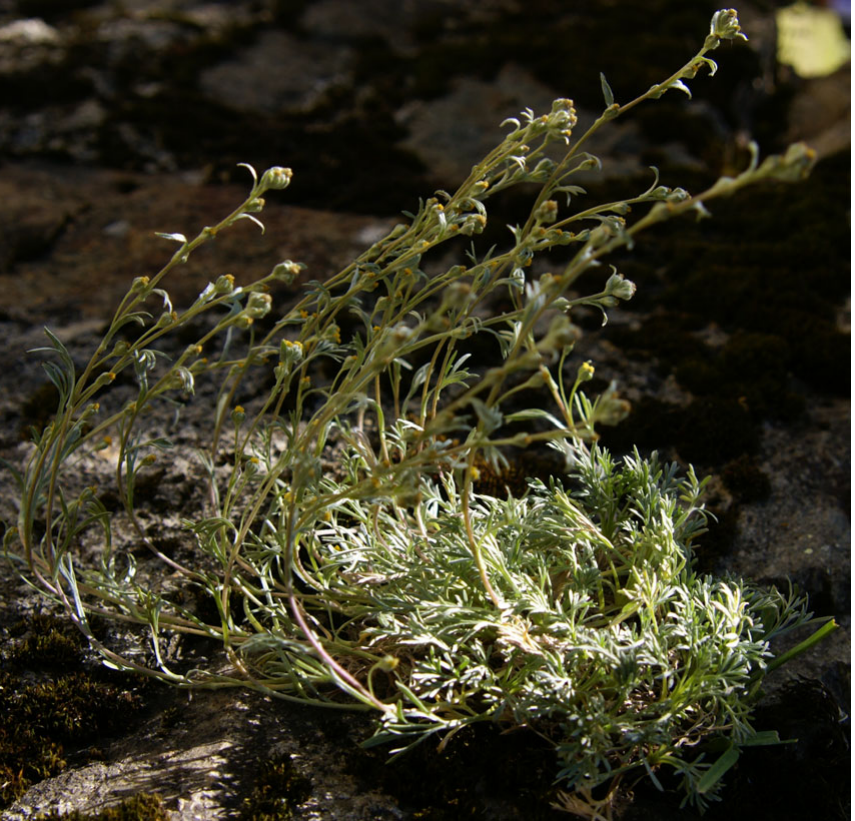
***Artemisia umbelliformis***.

*Artemisia umbelliformis *Lam. (Asteraceae) (Figure 7). Wild and cultivated. Genepì fumelo. Dried flowering aerial parts: macerated in alcohol as a digestive. Traded in the past and now (illegally). (CHI-GEF2) ++

*Artemisia vulgaris *(Asteraceae). Wild. Artumiso. Dried aerial parts: tea used as an emmenagogue (women) and to facilitate calving (cows). (CHI-ART) +++

*Avena sativa *L. (Poaceae). Cultivated in the past. Aveno. Fruit used as fodder for animals. +

*Boletus *spp. (Boletaceae). Wild. Boulet. Fruiting body: consumed (rare). +

*Brassica oleracea *L. (Brassicaceae). Cultivated. Chaoul. Fresh aerial parts: consumed in soups and as fodder for animals. Often harvested during the snow season and stewed with anchovies (*sancraou*). ++

*Bunium bulbocastanum *L. (Apiaceae). Wild. Gravaioun. Fresh tubers: eaten raw, or boiled, also in the dough of the *ravioles *(small dumplings made with flour, potatoes and alpine hut cheese, *tumo*). These uses are recalled as occurring only in the past. +

*Carlina acaulis *L. (Asteraceae). Wild. Chardousso. Fresh flower receptacle as a snack. Dried flowering aerial parts are hung on the outside door year round and used to forecast weather. (CHI-CHA1) ++

*Carum carvi *L. (Apiaceae). Wild. Chimmel, Serieis. Dried fruits: tea or macerated in alcohol as a digestive. Added to food as a seasoning (especially meat and poultry, particularly rabbit, or *sarass, a *local fermented and spiced ricotta cheese). (CHI-SER) +++

*Cetraria islandica *(L.) Acharius (Parmeliaceae). Wild. Gratacoul. Dried thallus: tea as a bechic. Traded in the past. (CHI-GRA) ++

*Chelidonium majus *L. (Papaveraceae). Wild. Erbo de Santo Mario. Fresh latex: topically applied for treating warts. (CHI-ESM) +

*Chenopodium bonus-henricus *(Chenopodiaceae). Wild. Bies, Zerbes, Orle (plural). Fresh young leaves: consumed boiled in soups and in omelettes. Also preserved in salt. Fodder for cows (reputed to be good as a "cleansing" agent). Topically applied for treating skin inflammation caused by stinging nettle. (CHI-BIE) +++

*Cirsium acaule *Scop. (Asteraceae). Wild. Vazanel. Fresh flower receptacle, consumed raw as a snack. +

*Cirsium arvense *(Asteraceae). Wild. Choussio. Fresh young leaves: boiled in soups. (CHI-CHO) +

*Cirsium spinosissimum *(L.) Scop. (Asteraceae). Wild. Chardousso. Fresh flower receptacles as a snack. Dried flowering aerial parts are hung on an outside door year round and used to forecast weather. (CHI-CHA2) ++

*Cynodon dactylon *(L.) Pers.(Poaceae). Wild. Gramoun. Roots: tea as a depurative. (CHI-GRA) +

*Echium italicum *L. (Borraginaceae). Wild. Erbo vieio. Fresh young leaves: boiled in soups. +

*Fagopyrum esculentum *Moench. (Polygonaceae). Cultivated in the past. Furmetin, Pignoulet. Fruits: ground into flour and used as the main ingredient in the local polenta, often prepared by alternating layers of polenta with layers of local cheese (*tumo*) and butter, or sometimes even adding potatoes and leeks. +++

*Fragaria vesca *L. (Rosaceae). Wild. Ambourses (plural), Mourso. Fruits: consumed as a snack. (CHI-MOU) ++

*Gentiana acaulis *L. (Gentianaceae). Wild. Chaousso de cucu. Dried leaves: tea as a digestive and anti-hypertensive. Dried flowers: macerated in alcohol or wine, as a digestive. (CHI-CHC) +++

*Gentiana lutea *L. (Gentianaceae). Wild. Ergensano. Dried roots (*reis*): macerated in wine, as a digestive, for "cleansing blood "/depurative, or "to make the blood thinner". Decoction used to enhance rumination in cows. (CHI-ERG) +++

*Heracleum sphodylium *L. (Apiaceae). Wild. Turmel. Fresh aerial parts: to be avoided as fodder (hay would remain humid and be of a very poor quality). (CHI-TUR) +

*Hordeum vulgare *L. (Poaceae). Cultivated in the past. Uerge.

Folk species: Brins, Brens. Fruits: crushed and used as fodder for hens; the bran used as fodder for cattle. Traditionally consumed as main ingredient in a particular kind of *polenta *(*poouties*). Folk species: Eplà, Pelá. Fruits: roasted and ground, in decoction, as a "coffee substitute". ++

*Hypericum perforatum *L. (Hypericaceae). Wild. Erbo di San Juan. Fresh flowering aerial parts: macerated in oil for treating burns. (CHI-ESJ) +

*Juglans regia *L. (Juglandaceae). Semi-domesticated. Nounzal (tree). Kernels: pressed to obtain the oil, which was commonly used in the past in the local cuisine. +

*Juniperus communis *L. (Cupressaceae). Wild. Chais. Dried fruits: used as a seasoning or macerated in grappa as a digestive. (CHI-CHI) ++

*Laburnum alpinum *(Mill.) Bercht. & J. Presl (Leguminosae). Wild. Albourn. Fresh flowering aerial parts (*bindareles*): put in the hen house to deter lice and flies. (CHI-ALB) ++

*Larix decidua *Mill. (Pinaceae). Wild. Melze, Merze. Resin: topically applied as a cicatrizing agent. (CHI-MEL) +++

*Lathyrus *sp. (Leguminosae). Wild. Lioum. Seeds: ground and the flour used in the past for preparing a kind of polenta. +

*Lens culinaris *Medik. (Leguminosae). Cultivated in the past. Lentie (plural). Seeds: in soups, or traditionally consumed as main ingredient in a particular kind of *polenta *(*poouties*). ++

*Linum usitatissimum *L. (Linaceae). Cultivated in the past. Lin. Seeds: as fodder for calves. Mixed with water, heated and externally applied on the chest for treating colds and coughs. +

*Malva neglecta *Wallr. and *M. sylvestris *L. (Malvaceae). Wild. Rioundelo. Fresh leaves: tea, also used in gargles, as an anti-inflammatory. Boiled in soups. (CHI-RIU) +++

*Mentha spicata *L. (Lamiaceae). Cultivated in home gardens. Mento. Fresh leaves: tea as a digestive. Also as a seasoning for pancakes (*bignes*). ++

*Myrrhis odorata *(L.) Scop. (Apiaceae). Wild. Charvei. Aerial parts: to be avoided as they are "poisonous". (CHI-CHV) +

*Narcissus poeticus *L. (Liliaceae s.l.). Wild. Susarelo, Joes de mel, Fior del mel. Flowers: sucked as a snack (especially by children in the past). (CHI-SUS) +

*Nasturtium officinale *R. Br. (Brassicaceae). Wild. Creissentin. Fresh leaves: consumed in salads. (CHI-CRE) +

*Nepeta cataria *L. (Lamiaceae). Wild. Erbo da chat. Dried aerial parts: tea as a mild tranquillizer. Traded in the past. +

*Onobrychis montana *DC. (Leguminosae). Wild. Sparsei. Fresh and dried aerial parts: highly valued fodder for animals. (CHI-SPA) +

*Petasites hybridus *(L.) P. Gaertn., B. Mey & Scherb. (Asteraceae). Wild. Chapus. Fresh leaves: fodder for hens. (CHI-CHP) ++

*Peucedanum ostruthium *(L.) W.D.J. Koch (Asteraceae). Wild. Algrot. Fresh root (*reis*): decoction or directly administered crushed and mixed with salt as a digestive (for cattle and sheep). (CHI-ALG) +++

*Pinus cembra *L. (Pinaceae). Wild. Elvio. Seeds: pressed to obtain oil (in the past). Cones: macerated in alcohol as a digestive. (CHI-ELV) +++

*Piper nigrum *L. (Piperaceae). Not local; bought in shops. Pepe. Dried fermented fruits: boiled in milk and butter for treating coughs. +

*Plantago lanceolata *L. and *P. major *L. (Plantaginaceae). Wild. Piantai. Dried leaves: tea as an appetizer and digestive (for calves only). Fresh leaves: topically applied with cream from cows' milk and bread or clay as a suppurative (also for cows, especially for treating inflamed hooves). (CHI-PIA1/CHI-PIA2) ++

*Polygonum bistorta *L. (Polygonaceae). Wild. Zonbuines (plural), Lingo boino, Linboina. Fresh young leaves: consumed in soups. (CHI-ZON) +++

*Prunus avium *L. (Rosaceae). Semi-cultivated. Serieses (fruits, pl.). Fresh fruits: consumed raw as a snack. +

*Prunus domestica *L. (Rosaceae). Semi-cultivated. Brigne (fruits, pl.). Fresh fruits: consumed raw. +

*Rosa canina *L. (Rosaceae). Wild. Bouchier, Bouchiet. Dried pseudofruits: tea as an anti-diarrhoeic. Ground and added to bread dough in the past. (CHI-BUC) ++

*Rubus ideaus *L. (Rosaceae). Wild. Amurse (plural). Fresh fruits: eaten raw or processed into home-made jams. +++

*Rumex acetosa *L. and *R. acetosella *L. (Polygonaceae). Wild. Setou. Fresh leaves. eaten raw as a snack. (CHI-SET1 and CHI-SET2) +

*Rumex alpinus *L. (Polygonaceae). Wild. Gravasso, Ravasso. Fresh leaves: topically applied as a cicatrizing agent. Fresh leaves and stems: fodder for cattle. Fresh stems: eaten raw with salt as a snack or boiled like asparagus; occasionally used in soups (especially in the past). (CHI-GRA) ++

*Sambucus nigra *L. (Caprifoliaceae). Wild. Sambuc. Fresh fruits: wine and jams (only in the lower part of the studied area). +

*Secale cereale *L. (Poaceae). Cultivated in the past. Seil. Fruits: flour. Widely used in the past as main ingredient for bread, and also for the local home-made noodles, *courzeitin*, and a porridge-like preparation based on milk and butter, *panado*. +++

*Sedum album *L. (Crassulaceae). Wild. Salabron. Fresh aerial parts: topically applied for treating skin inflammation caused by stinging nettle. (CHI-SAL) +

*Solanum tuberosum *L. (Solanaceae). Cultivated. Trufes (plural). Fresh tubers: consumed boiled; traditionally added to the dough of the local dumplings (*ravioles*). +++

*Sorbus aria *(L.) Cratz. (Rosaceae). Wild. Alier. Fresh fruits (*alìe*, pl.): eaten after natural fermentation on straw. Once dried, ground and added to bread dough (in the past). +

*Sorbus aucuparia *L. (Rosaceae). Wild. Pizzera. Fresh fruits: to be avoided by humans but appreciated by wild birds. +

*Tanacetum vulgare *L. (Asteraceae). Wild and semi-domesticated. Archebuse. Fresh leaves: macerated in alcohol as digestive. (CHI-ARC) +++

*Taraxacum officinale *Weber (Asteraceae). Wild. Mourioun. Fresh leaves: consumed in salads or boiled. (CHI-MOU) +++

*Thymus pulegioides *L. s.l. (Lamiaceae). Wild. Serpour, Serpoul. Dried leaves: tea as a digestive, for treating sore-throats, or as a diuretic. As a seasoning in the local cuisine (especially the local, home-made, fermented and spiced ricotta: *sarass*). (CHI-SER) ++

*Tragopogon pratensis *L. (Asteraceae). Wild. Barbouch, Barbabouch. Fresh flower buds and young leaves: consumed raw or boiled. (CHI-BAR) +++

*Tussilago farfara *L. (Asteraceae). Wild. Pupettes. Dried flowers: tea as a bechic. Traded in the past. (CHI-PUP) +

*Urtica dioica *L. and other *U*. species (Urticaceae). Wild. Urtio. Fresh leaves: consumed in soups or frittata. Tea as a diuretic (mainly for women; men should take it in limited amounts only.). (CHI-URT) +++

*Vaccinium myrtillus *L. (Ericaceae). Wild. Aize. Fresh fruits: consumed raw or processed in jams or cordials. +++

*Vaccinium uliginosum *L. (Ericaceae). Wild. Poumarette (plural). Fresh fruits: often mixed with *V. myrtillus *fruits, consumed raw or processed in jams or cordials (less popular than *V. myrtillus *fruits) ++

*Vaccinium vitis-idaea *L. (Ericaceae). Wild. Petemerlet. Fresh fruits: raw as a snack. ++

*Valerianella *sp. (Valerianaceae). Wild. Salzet. Fresh young leaves: consumed in salads. (CHI-SAL) +

*Veratrum album *L. (Liliaceae s.l.). Wild. Varaire. Roots: topically applied as an anti-lice treatment for calves. (CHI-VAR) +

*Verbascum nigrum *L. (Schrophulariaceae). Wild. Nevioun. Flower: tea as a bechic. (CHI-NEV) +

*Veronica allionii *Vill. (Schrophulariaceae). Wild. Jaspertero. Dried flowering, aerial parts: tea (use not recalled). Decoction given to cows (use not recalled). Traded in the past. (CHI-JAS) +++

*Veronica beccabunga *L. (Schrophulariaceae). Wild. Seiraset. Fresh leaves: consumed raw in salads. (CHI-SER) +

*Vicia faba *L. (Leguminosae). Cultivated in the past. Faves (plural). Seeds: traditionally consumed as main ingredient of a particular kind of *polenta *(*poouties*). +

*Viola odorata *L. (Violaceae). Wild. Vioulette (plural). Dried leaves: tea for treating colds and coughs (low dosage). Fresh leaves: boiled in soups. Traded in the past. (CHI-VIO) ++

*Vitis vinifera *L. (Vitaceae). Vinegar. Purchased in the surrounding lower areas. Poultice mixed with clay: externally as a suppurative. ++

*Zea mais *L. (Poaceae). Cultivated. Meliga. Seeds: ground into flour and used as main ingredient for a particular kind of local polenta. +++

Unidentified taxon. Wild. Erbo dousso. Fresh aerial parts. Boiled in soups. +

Unidentified taxon. Wild. Gialinettes, Gialinettes grasses (plural). Fresh aerial parts: boiled in soups. ++

Unidentified taxon. Wild. Ontu. Root: decoction given to calves to treat digestive problems. +

## Discussion

### Is the Occitan TK of gastronomic and medicinal plants different from other Western Alpine TK systems?

**Figure 8:**
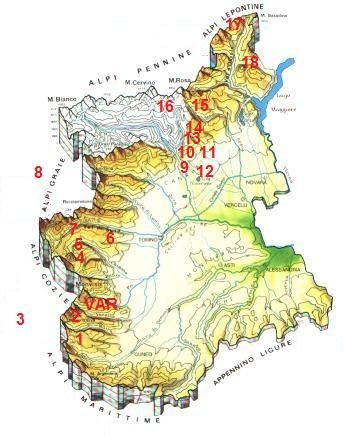
**Location of the Western Alpine sites, where previous ethnobotanical studies have been carried out (numbers correspond to those used in the first column of Table 1; VAR: study area - upper Varaita valley)**.

Table 1 shows a comparison between the medico-ethnobotanical data collected in the study area and data gathered from previous ethnobotanical studies conducted in other Alpine valleys in Piedmont and surrounding regions (figure 8) [15-26]. The data available in the literature on Piedmontese ethnobotany are not methodologically consistent, and the field methods used in early studies have only rarely been properly described, nevertheless the descriptive comparison shows that approximately one fourth of the quoted taxa in our study occur in the TK systems of other Occitan valleys. One exception, however, is represented by the ethnobotanical data of two Occitan valleys whose inhabitants are Waldensians. This group shows an ethnobotanical knowledge that seems to be very distinct from that of the bulk of all other Occitan areas.

**Table 1:** Comparison between the folk medicinal taxa quoted in the upper Varaita valley and those quoted in other ethnobotanical studies conducted in the past in the Western Alps (see Figure 8).

Area	Year of publication of the field ethnobotanical study	Ethnic/cultural group(s)	Recorded folk medicinal taxa	Medicinal taxa also used in the Upper Val Varaita (VAR)
Ubaye Valley (1)	1990	OC	112	25
Maira Valley (2)	1982	OC	127	24
Sangone Valley (6)	1977	FP	72	24
Stura Valley (1)	2006	OC	102	23
Susa Valley (7)	1977	OC and FP	99	20
Mastallone Valley (Rimella) (11)	1957	WA	25	15
Les Allues (8)	1986	FP	27	13
Formazza Valley (17)	1955	WA and PE	27	12
Grande Valley (9)	1957	WA and PE	23	11
Sermenza Valley (10)	1957	WA and PE	21	11
Strona Valley (12)	1957	PE	25	11
Mastallone Valley (Forbello) (11)	1957	PE	32	10
Anzasca Valley (13)	1955	WA and PE	27	10
Bognanco Valley (15)	1955	PE	14	8
Vigezzo Valley (18)	1955	PE and LO	20	8
Antrona Valley (14)	1955	PE and LO	13	7
Valtournanche (16)	1983	FP	64	6
Chisone Valley (5)	1984	OC*	35	2
Germanasca Valley (4)	1984	OC*	19	0

Numbers in the first column correspond to those used in Figure 2.

LO: Lombard. FP: Franco-Provençal. OC: Occitan. OC*: Occitan and Waldensian (as per religion). PE: Piedmontese. WA: Walser

### Genepì: from an herbal tea to a gastronomic specialty

The term genepì/génépì (synonyms: génepi, génipi) is applied in the Western Alps (in Occitan, Franco-Provencal, and also in French and Italian too) to diverse species of locally growing Alpine wormwood (esp. *Artemisia genipi, A. glacialis, A. umbelliformis*). This term has probably Savoyard origins, and is derived from the Latin *Dianae spicum *(*épi de Diane *in French, *Diana's ear *in English) [41].

The ethnobotany of *genepì *in the Western Alps has never been recorded specifically or systematically, although scattered notes have occurred in Alpine ethnobotanical studies over the last few decades (Table 2). From these brief ethnographic reports we can conclude that genepí was originally used for preparing herbal teas, and shepherds in the mountainous areas frequently used to decoct the aerial parts in milk and butter instead of water. Following the introduction of wine and grappa (purchased from farmers living in the hills and on the plains), the herb probably was macerated in alcohol as its use as a liqueur began to appear over a century ago in different gastronomic and commodity treatises in France and Italy [42-45].

**Table 2:** Folk medical uses of the aerial parts of Alpine wormwood (*Artemisia genipi, A. glacialis, and A. umbelliformis*) in diverse North-Western Alpine areas (See Figure 8 to located most of these areas on the map).

Area(s)	Ethnic/cultural group(s)	Preparation(s)	Folk medical use(s)
Anniviers Valley, Switzerland	FP	Macerated in alcohol; made into home-made distillate with chopped apples and sugar.	Depurative, digestive; high doses are considered dangerous (inducing circulatory disturbances in eyes and nervous system)
Aosta Valley, Italy	FP	Infusion in milk with honey	Anti-tussive, anti-cold
Grande and Formazza valleys, Italy	WA and PE	Infusion in water; decoction in milk or wine; macerated in alcohol	Anti-tussive, tonic, digestive
Les Allues, France	FP	Infusion in water or milk and butter (with sugar added); on rare occasions macerated in alcohol	Anti-cold, panacea; high doses are considered dangerous
Sangone Valley, Italy	FP	Infusion in water	Digestive
Susa Valley, Italy	OC and FP	Infusion in water	Digestive
Germanasca and Chisone valleys, Italy	OC*	Infusion in water	Digestive
Maira Valley, Italy	OC	Infusion in water; macerated in alcohol	Diaphoretic, to prevent mountain sickness and syncopal attacks

FP: Franco-Provençal. OC: Occitan. OC*: Occitan and Waldensian (according to religion).

PE: Piedmontese. WA: Walser

This seems to concur with what has been hypothesized in other Italian Alpine regions regarding herbal macerates in *grappa *[46], which they were considered sometimes traditional "medicinal" preparations. Preparation of liqueurs began to be relevant especially in the 1960's, when Italy began to enjoy better economic conditions which enabled isolated rural communities to have at their disposal larger amounts of grappa and/or industrial alcohol.

This shift is quite interesting since it demonstrates, not only the overlapping between food and medicine, but also how the preponderant role of one of the two domains can be the result of dynamic historical, social, and economic changes.

Nowadays, *A. umbelliformis *is widely cultivated in the Western Alps to supply the local distilleries that produce the alcoholic macerate of *genepì*, which widely traded all over the world. Because of this economic interest, local institutions in Piedmont are very active in putting in place measures for improving the quality and yield of cultivated Alpine wormwood [47].

All our informants, however, were very keen to underline the difference in taste between the industrial genepì (produced from the aerial parts of *A. umbelliformis *only - the only *Artemisia *species of the three that has been and can be cultivated with substantial agronomic results) and the home-made liqueur (which generally always contain *A. genipi *or sometimes a mix of *A. genipi *and *A. glacialis*). According to locals in the upper Varaita valley, the specific occurrence in the recipe of *A. genepì *(locally defined as the "male genepì") is crucial for achieving a superb tasting alcoholic beverage.

### Collection of local herbs from the wild: sustainable perspectives for the future

*A. genipi *grows in the study area only between 2600-2700 meter a.s.l., and it is very difficult to find, which is why it is so highly valued. Although the species is endangered and its collected is prohibited in many Alpine areas, in the Piedmontese legislation the plant is not included in the regional Red List and aerial parts corresponding to up to 5 plants per person can be gathered [48].

Locals in the upper Varaita valley continue to gather the flowering tops (locally named "ears") of *A. genipi *during July and August, to dry them, and them macerate them in alcohol for at least a month and a half (the alcoholic macerate is then diluted with sugar and water, and filtered). In the past this species was probably heavily exploited by the locals in Chianale, who used to sell it (generally dried) to local distilleries both on the plain of Piedmont and on the French side (often smuggled across secondary pathways over the Alps that were passable with donkeys only). Informants in Chianale recalled how this activity provided the inhabitants with their most important income from a few decades after World War II. There is probably still some illegal gathering of this species that provides additional cash to the people of the mountain. A few informants told us that the income that can be derived from gathering genepì (*A. genipi *and - to lesser extent - *A. glacialis*) can reach 15-20 thousand Euros every summer for one single household.

In table 3 we report on the current legal framework [48-50], the estimated ecological availability and potentialities for the local market of the most quoted, traditionally used wild herbs in the study area.

**Table 3:** Local market potential of the most quoted wild gastronomic and medicinal herbs (excluding wild berries) in the upper Varaita valley

Species	Estimated ecological availability in the upper Varaita valley	Gathering permitted in the Cuneo Province	Estimated potential for the local market (details)
***Achillea herba-rotta *(herb)**	Medium	Aerial parts corresponding to five plant samples; up to 1 kg (dried aerial parts) if special permission is obtained from local authorities.	**High** **(dried herb to be sold as a tea).**
***Arnica montana*** **(flowers)**	Medium	Aerial parts corresponding to five plant samples; up to 5 kg (dried flowers/roots) if special permission is obtained from local authorities.	**High** **(dried flowers to be sold as a tea).**
*Artemisia absinthium* (herb)	High	Aerial parts corresponding to five plant samples; up to 2 kg (dried aerial parts). if special permission is obtained from local authorities.	Low (dried herbs to be sold as a tea); Medium (ingredient of home-made digestives).
*Artemisia genipi, A. glacialis, A. umbelliformis* (aerial parts and flowers)	Low	Aerial parts corresponding to five plant samples; up to 1 kg of each species (dried aerial parts). if special permission is obtained from local authorities.	Very low, given the limited ecological availability.
***Carum carvi*** **(fruits)**	Medium-high	Not clear: aerial parts corresponding to five plant samples or unlimited gathering. Unlimited gathering, due to the fact that the plant can be considered "commonly consumed" in the study area.	**High (dried fruits as a tea or seasoning).**
***Chenopodium bonus-henricus *(leaves)**	Very high	Unlimited gathering, due to the fact that the plant can be considered "commonly consumed" in the study area.	**High (as a preserved vegetable?)**
*Gentiana acaulis* (leaves and flowers)	Medium-low	Aerial parts corresponding to five plant samples	Low (as an ingredient for digestives) due to the limited availability in the area.
***Gentiana lutea*** **(roots)**	High	Up to 10 kg (dried roots), if special permission is obtained from local authorities.	**High (dried root as a tea or ingredient for digestives).**
*Malva *spp.	High	Unlimited gathering, due to the fact that the plant can be considered "commonly consumed" in the area.	Medium (as a tea or as a preserved vegetable).
*Peucedanum ostruthium* (roots)	Medium-low	Up to 2 kg (dried roots), if special permission is obtained from local authorities.	Low (as a tea).
*Pinus cembra* (cones)	Low	Theoretically unlimited.	Low (as an ingredient for digestives) due to the limited availability.
*Polygonum bistorta* (leaves)	High	Unlimited gathering, due to the fact that the plant can be considered "commonly consumed" in the area.	Medium (as a preserved vegetable).
*Rumex alpinus* (leaves/stalks)	Medium	Unlimited gathering, due to the fact that the plant can be considered "commonly consumed" in the area.	Low (as a preserved vegetable).
***Tanacetum vulgare*** **(leaves)**	Medium	Aerial parts corresponding to five plant samples; up to 5 kg (dried aerial parts) if special permission is obtained from local authorities.	**High (as a tea or as an ingredient for digestives).**
***Taraxacum officinale*** **(leaves)**	High	Unlimited gathering, due to the fact that the plant can be considered "commonly consumed" in the area.	**High (as a preserved vegetable or a tea).**
***Tragopogon pratensis*** **(leaves and flower buds)**	Medium	Unlimited gathering, due to the fact that the plant can be considered "commonly consumed" in the area.	**High (as a preserved vegetable).**
*Urtica *spp. (leaves)	High	Unlimited gathering, due to the fact that the plant can be considered "commonly consumed" in the area.	Low (as a preserved vegetable).
*Veronica allionii* (flowering aerial parts)	Low	Aerial parts corresponding to five plant samples.	Low (as a tea) due to the limited availability in the area.

It is clear that further exploitation of the *genepì *species cannot be recommended, however sustainable gathering of other local plant resources (wild vegetables and herbal teas) that are widely available in the area, could be a very interesting way to complement the eco-touristic activity, that is quite exclusively concentrated during the summer months, thereby providing an important source of income for the few remaining inhabitants.

## Conclusion

Traditional food and medical uses of 88 local plants were recorded in the upper part of the Varaita valley. Analytical comparisons with other ethnobotanical studies previously carried out in other Piemontese and surrounding areas have shown that: a) traditional knowledge has been heavily eroded in the Varaita valley, and b) approximately one fourth of the quoted botanical taxa were also recorded in earlier surveys conducted in other Occitan valleys in the Italian Alps. Furthermore, the continued widespread local collection from the wild of the aerial parts of Alpine wormwood for the preparation of liqueurs should be seriously reconsidered in terms of sustainability, given the limited availability of these species even though this collection is culturally salient in the entire study areas, On the other hand interesting potentialities for the local market could be tested for other widely available wild species, such as *Achillea herba-rotta, Arnica montana, Carum carvi, Chenopodium bonus-henricus, Tanacetum vulgare, Taraxacum officinale*, and *Tragopogon pratensis*.

## Competing interests

The authors declare that they have no competing interests.

## Authors' contributions

AP and MEG have collected the data in the field; AP has analyzed the data and drafted the conclusions. All authors read and approved the final manuscript.
